# The bug, the burden, and the biology: beyond host-centric phenotyping in sepsis

**DOI:** 10.1172/JCI203658

**Published:** 2026-03-16

**Authors:** Georgios D. Kitsios, Rebecca M. Baron

**Affiliations:** 1 Division of Pulmonary, Allergy, Critical Care and Sleep Medicine and; 2 Acute Lung Injury and Infection Center, University of Pittsburgh, Pittsburgh, Pennsylvania, USA.; 3 Division of Pulmonary and Critical Care Medicine, Department of Medicine, Brigham and Women’s Hospital and Harvard Medical School, Boston, Massachusetts, USA.

## Abstract

For over a decade, sepsis phenotyping has identified hyperinflammatory and hypoinflammatory subphenotypes using host biomarkers and clinical variables, without factoring in contributions from infectious insults across patients. In this issue, Chanderraj and colleagues challenge this host-centric paradigm by demonstrating that pathogen characteristics independently contribute to sepsis subphenotypes. They reported that *Enterobacterales* infections, particularly *Escherichia coli*, strongly associated with hyperinflammatory subphenotypes, independent of illness severity. Bacterial burden, anatomic barrier breach, and circulating pathogen-associated molecular patterns influence phenotypic classification, with implications extending to culture-negative sepsis. Animal models supported causality, while reanalysis of an observational cohort and a clinical trial revealed that lactate clearance’s prognostic value and therapeutic effects of endotoxin removal with polymyxin B hemoadsorption vary by subphenotype and pathogen. These findings lay groundwork for integrative host-pathogen phenotyping; for precision medicine in critical illness, we must know not only *who* is sick, but *what* made them sick, and *how* the two interact.

## Introduction

**Figure 1:**
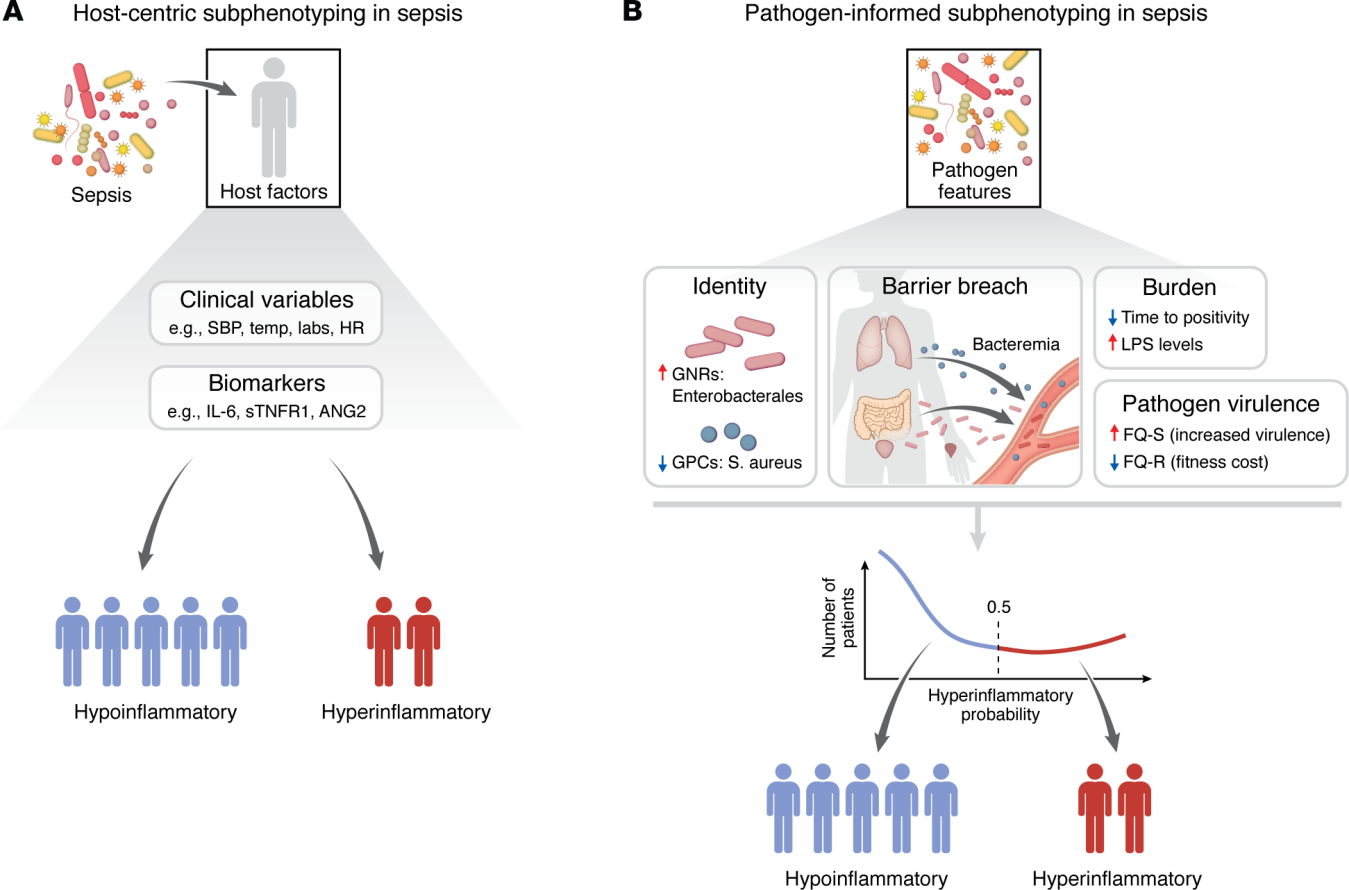
Integrative host-pathogen phenotyping in sepsis: from host-centric to pathogen-informed models. (**A**) Traditional host-centric subphenotyping in sepsis assumes a relatively uniform microbial insult, with phenotypic heterogeneity attributed primarily to host factors. Patients are dichotomized into hyperinflammatory (red) or hypoinflammatory (blue) states based on clinical variables (such as systolic blood pressure [SBP], body temperature [temp], laboratory testing results, and heart rate [HR]) and circulating biomarkers (such as IL-6, soluble [s]TNFR1 and ANG2). Hyperinflammatory patients have higher severity of illness as measured by organ dysfunction, and worse clinical outcomes compared with hypoinflammatory patients, with potentially differential responses to treatments and interventions. (**B**) Pathogen-informed subphenotyping, as reported by Chanderraj et al. (4), incorporates microbial features that shape host inflammatory responses. Illustrated dimensions include pathogen identity (e.g., gram-negative rods (GNRs) such as *Enterobacterales* versus gram-positive cocci (GPCs) such as *S*. *aureus*); burden (e.g., time-to-positivity, lipopolysaccharide levels); barrier breach and anatomic source (i.e., bacteremia from gastrointestinal or respiratory sources); and pathogen virulence or fitness traits (e.g., fluoroquinolone-resistance [FQ-R] or -sensitivity [FQ-S]). These factors jointly influence the probability of hyperinflammatory host response, represented as a continuous spectrum rather than a binary state, with red upward arrows and blue downward arrows illustrating the impact of pathogen features on hyperinflammatory probability. In this framework, distinct pathogen characteristics shift patients toward higher or lower hyperinflammatory probabilities, emphasizing sepsis as a dynamic host-pathogen interaction rather than a fixed host-defined phenotype.

Research on critical illness and sepsis phenotyping has parsed observable heterogeneity using unsupervised methods applied to diverse data types, including transcriptomics, plasma biomarkers, and clinical variables (1–3). Despite methodological differences, recurring biological patterns emerge: hyperinflammatory states with high innate immune activation and coagulopathy; immunoadaptive versus immunosuppressed profiles; and coagulopathic/vasoplegic endotypes with endothelial injury. High-risk groups cluster around overwhelming inflammation-coagulopathy and/or impaired adaptive immunity (1). Yet these prognostically robust schemes have operated on an implicit assumption: that infectious insults are essentially equivalent across patients, with phenotypic heterogeneity arising from interindividual differences in host response to those insults (Figure 1A). The focus has been on *how* patients differ in response to infection, rather than on *what* differs in the infections themselves. What if this assumption is incomplete? What if subphenotypes also reflect different pathogens, varying in identity, burden, and virulence?

In this issue of the Journal, Chanderraj and colleagues (4) challenged this host-centric paradigm by systematically examining what has been incompletely integrated into subphenotyping frameworks: the bug, the burden, and the biology of the pathogen itself. Their findings represent a conceptual shift from viewing sepsis heterogeneity as purely host-driven toward understanding it as an integrated product of host-pathogen interactions.

## The bug: pathogen identity matters

Chanderraj et al. analyzed 8,280 patients who were critically ill with sepsis, classifying them into subphenotypes using a validated clinical variable–based machine learning model: 22% fell into a hyperinflammatory phenotype characterized by higher illness severity (per APACHE IV scores), greater duration of shock, and higher 90-day mortality, while 78% classified as a hypoinflammatory phenotype (4). Among 1,350 patients with monomicrobial bacteremia, pathogen identity was strongly associated with subphenotype, and this association was two orders of magnitude stronger than with illness severity. Pathogen identity predicted which subphenotype a patient expressed far more consistently than prediction based on illness severity, suggesting that hyper- versus hypoinflammatory subphenotypes more strongly reflected pathogen-driven biology than host factors alone.

The specificity of pathogen identity for subphenotype was striking. Infection with *Enterobacterales* species — particularly *E*. *coli* and *Klebsiella spp* — was independently associated with the hyperinflammatory subphenotype. Patients with *E*. *coli* bacteremia had a median hyperinflammatory probability of 0.50 versus 0.14 for *Staphylococcus aureus*, with gram-negative bacteremia overall showing higher probabilities (0.43) than gram-positive bacteremia (0.12).

These *Enterobacterales* associations extended beyond bacteremia to urinary and respiratory tract infections, though with smaller effect sizes (median probabilities 0.10-0.12 vs. 0.50 for bacteremia), suggesting that barrier breach and bloodstream invasion amplify hyperinflammatory responses beyond pathogen identity alone.

## The burden and the barrier breach

The authors demonstrated that bacterial burden (measured by time to blood culture positivity) was independently associated with hyperinflammatory classification. Shorter time to positivity, reflecting higher bacterial load, strongly predicted the hyperinflammatory subphenotype (mean 12.9 hours versus 23.9 hours, *P* < 0.001), with each hour of delay reducing the odds of hyperinflammatory classification (4).

Pathogen burden in blood may reflect not only the quantity of replicating organisms but also the anatomic barrier breached. Among patients with *E*. *coli* bacteremia, intraabdominal sources showed the highest hyperinflammatory probabilities, while for *S*. *aureus* bacteremia, pneumonia emerged as the highest-risk source — recapitulating observations that double-positive (blood and respiratory cultures) pneumonias portend worse outcomes (5). These patterns suggest that breach of certain anatomic barriers — particularly the gut epithelium and alveolar-capillary membrane — may permit high-burden bacteremia, amplifying the hyperinflammatory response (6).This burden-barrier framework extends beyond culture-positive cases that were the main focus of Chanderraj et al.’s study. In the EUPHRATES trial population, circulating endotoxin (lipopolysaccharide, LPS) activity correlated positively with hyperinflammatory probability, even when 70% of patients had negative blood cultures (7). Our group and others have observed that higher concentrations of plasma microbial cell–free DNA correlate with systemic inflammation, severity of organ dysfunction, and adverse outcomes in sepsis and pneumonia (8–10). Similarly, circulating β-D-glucan — a fungal cell wall component — has been associated with the hyperinflammatory subphenotype, even in patients without documented fungal infection (11). These culture-independent measures demonstrate that the amount of circulating pathogen–associated molecular patterns (PAMPs, including endotoxin, microbial DNA, β-D-glucan) can influence systemic host responses. Importantly, in culture-negative or nonbacteremic sepsis, circulating PAMPs may originate not only from a primary pathogen but also from barrier disruption that permits translocation of colonizing organisms or their fragments, emphasizing the need to study pathogen biology and mechanisms of host barrier disruption in parallel (12)

The gut warrants particular attention as a source of both live pathogens and PAMPs (13). Circulating DNA from *Enterobacterales* has been associated with the hyperinflammatory subphenotype (10), and intestinal domination by *Enterobacterales* species precedes bacteremia in immunocompromised hosts (14, 15). Host factors, including surfactant protein D expressed in the gallbladder and secreted into the gut, modulate *E*. *coli* colonization and mortality during abdominal sepsis (16), while host-pathogen interactions at the gut-luminal surface influence intestinal barrier integrity (17). This convergence of gut domination by *Enterobacterales* leading to barrier breach with high-burden bacteremia likely explains why intraabdominal *E*. *coli* infections associate with the highest hyperinflammatory probabilities.

## The biology: mechanisms and clinical implications

Beyond pathogen identity and burden, Chanderraj et al. examined virulence as a determinant of subphenotypes (4). Using fluoroquinolone resistance as a proxy for fitness cost in *Enterobacterales* species, they found that fluoroquinolone-resistant isolates — which exhibit reduced virulence in vitro — were associated with decreased probability of the hyperinflammatory subphenotype (0.14 versus 0.57, *P* < 0.001). This observation underscores that not all *Enterobacterales* infections are immunologically equivalent. More broadly, the biological basis for *Enterobacterales’* association with hyperinflammation may involve LPS, a potent TLR4 activator whose structure — particularly the lipid A moiety — can modulate immunogenicity by up to several orders of magnitude (18).

On the host side, hyperinflammatory probability correlated more strongly with procalcitonin, aligning with prior latent class analyses (19). Importantly, lactate clearance (20) conferred stronger survival benefit in hyperinflammatory patients, with similar effects in *Enterobacterales* bacteremia. Chanderraj et al.’s reanalysis of the EUPHRATES trial revealed significant treatment effect modification; specifically, Polymyxin B hemoadsorption — which removes endotoxin — was associated with statistically significant harm in patients who were hypoinflammatory, while showing no benefit in patients who were hyperinflammatory (4). Together, these findings suggest that pathogen features impact inflammatory intensity and host metabolism; however, predicting treatment response may ultimately require integration of both pathogen characteristics and host response profiling.

Controlled animal models corroborated these observations (4). In swine, *E*. *coli* sepsis with direct lung injury provoked higher hyperinflammatory probabilities compared with lung injury alone. In murine peritonitis using two *Enterobacterales* species, increasing bacterial inoculum dose-dependently elevated inflammatory biomarkers (sTNFR1, IL-6, CXCL1). While these models have inherent limitations, including use of artificial inocula, direct organ inoculation, and species differences, they support the directionality of the authors’ thesis: pathogen characteristics causally influence the development of hyperinflammatory responses. These and other translational studies remain essential to understanding host-pathogen heterogeneity and identifying therapeutic targets (16, 21), even if they cannot fully recapitulate the complexity of human sepsis.

Despite the meaningful findings presented in this study, mechanism remains incompletely defined, and questions remain. Do differences in LPS structure explain the specificity observed? Does host genetic variation in pattern recognition receptors modulate responsiveness? Are pathogen characteristics altered by interaction with host-derived proteins? Are other PAMPs, such as flagellin or peptidoglycan, contributing? What is the influence of the microbiome on specific pathogens and host responses upon presentation and following broad-spectrum antibiotic therapy or stress-dose steroids? Future studies integrating microbiota profiles, dominant pathogen genomics, host receptor genetics, and functional immunology will be essential. Notably, pathogen-informed subphenotypes may represent mechanistically distinct endotypes, i.e. biological disease variants defined by pathogen-specific triggering of host pathways. If *E*. *coli*–driven hyperinflammation operates through different molecular mechanisms than *S*. *aureus*–driven responses, these represent true endotypes rather than simply prognostic clusters, with implications for targeted therapeutic development.

## The boundaries: limitations of pathogen-informed subphenotyping in sepsis

This work has important limitations that define future priorities. First, culture-positive bacteremic sepsis represents only 10%–15% of community-onset sepsis cases (22). While endotoxin analysis and prior culture-independent microbial profiling work suggest these findings generalize to culture-negative sepsis (8–10), prospective validation integrating both culture-based and culture-independent diagnostics is needed (9, 21).

Second, pathogen characteristics explain only modest variance in subphenotype classification. Host factors such as genetics, comorbidities, immune history, and microbiome composition may also be important. Pathogen features are not deterministic; rather, they act as proximal triggers whose effects depend on host capacity to contain or amplify inflammation. Moreover, pathogens interact with complex microbial communities, such that sepsis often reflects polymicrobial dynamics rather than single-pathogen biology. (12).

Third, clinical variable–based classifiers may have up to 22% misclassification versus biomarker models (23). While clinical models offer accessibility, targeted immunomodulation trials may require the biological fidelity of biomarker models.

Fourth, these subphenotypes evolve dynamically, with hyperinflammatory probabilities declining over the ICU course (24, 25). Whether these transitions reflect pathogen clearance, or whether persistent or recurrent infections prevent recovery, remains unknown. Longitudinal studies integrating microbial burden with subphenotype trajectories could identify optimal timing for interventions.

Finally, mechanistic pathways require further interrogation. Beyond PAMPs and pattern recognition receptors, pathogen-derived metabolites may shape host responses: acetylated polyamines produced by gram-negative bacteria during bloodstream infection influence both bacterial virulence and host metabolism (21). Addressing these questions requires deeply phenotyped cohort studies with serial host and pathogen sampling, particularly from abdominal sources of sepsis. The majority of accumulated cohorts reflect predominantly respiratory sepsis (26), yet, abdominal-derived pathogens appear critical to hyperinflammatory responses (4).

## The bold road ahead: integrative host-pathogen phenotyping

Chanderraj and colleagues establish that pathogen characteristics — identity, burden and virulence — are independent predictors of sepsis subphenotypes, moving beyond the assumption that subphenotypes reflect only host heterogeneity (Figure 1B). Future clinical trials should integrate pathogen diagnostics and host responses into enrollment and stratification schemes. Platform trials, such as the PANTHER network for acute respiratory distress syndrome (ARDS) (27), offer a framework for testing subphenotype-targeted and pathogen-informed interventions simultaneously. To realize precision medicine in critical illness, we need to know not only *who* is sick, but *what* made them sick, and *how* the two interact. This study brings us meaningfully closer to that goal.

## Funding support

This work is the result of NIH funding, in whole or in part, and is subject to the NIH Public Access Policy. Through acceptance of this federal funding, the NIH has been given a right to make the work publicly available in PubMed Central.

- NIH (R01 176668; to GDK).

## Version 1 03/16/2026

Electronic publication
